# Preliminary Evaluation of the Effect of Mechanotactile Feedback Location on Myoelectric Prosthesis Performance Using a Sensorized Prosthetic Hand

**DOI:** 10.3390/s22103892

**Published:** 2022-05-21

**Authors:** Eric D. Wells, Ahmed W. Shehata, Michael R. Dawson, Jason P. Carey, Jacqueline S. Hebert

**Affiliations:** 1 Department of Mechanical Engineering, University of Alberta, Edmonton, AB T6G 2R3, Canada; jpcarey@ualberta.ca; 2 Division of Physical Medicine and Rehabilitation, Department of Medicine, University of Alberta, Edmonton, AB T6G 2R3, Canada; ahmed.shehata@ualberta.ca (A.W.S.); mrd1@ualberta.ca (M.R.D.); jhebert@ualberta.ca (J.S.H.); 3 Glenrose Rehabilitation Hospital, Alberta Health Services, Edmonton, AB T5G 0B7, Canada

**Keywords:** prosthesis, grip force, compliant, feedback, somatotopical, modality-matched, capacitive, tactile sensor, inexpensive

## Abstract

A commonly cited reason for the high abandonment rate of myoelectric prostheses is a lack of grip force sensory feedback. Researchers have attempted to restore grip force sensory feedback by stimulating the residual limb’s skin surface in response to the prosthetic hand’s measured grip force. Recent work has focused on restoring natural feedback to the missing digits directly through invasive surgical procedures. However, the functional benefit of utilizing somatotopically matching feedback has not been evaluated. In this paper, we propose an experimental protocol centered on a fragile object grasp and lift task using a sensorized myoelectric prosthesis to evaluate sensory feedback techniques. We formalized a suite of outcome measures related to task success, timing, and strategy. A pilot study (*n* = 3) evaluating the effect of utilizing a somatotopically accurate feedback stimulation location in able-bodied participants was conducted to evaluate the feasibility of the standardized platform, and to inform future studies on the role of feedback stimulation location in prosthesis use. Large between-participant effect sizes were observed in all outcome measures, indicating that the feedback location likely plays a role in myoelectric prosthesis performance. The success rate decreased, and task timing and task focus metrics increased, when using somatotopically-matched feedback compared to non-somatotopically-matched feedback. These results were used to conduct a power analysis, revealing that a sample size of *n* = 8 would be sufficient to achieve significance in all outcome measures.

## 1. Introduction

Upper limb amputation results in loss of both motor and sensory function of the hand, negatively impacting an individual’s economic, psychological, and social well-being [1]. Prosthetic technology attempts to mitigate these effects by partially restoring functionality to the lost limb. Recent research in the area focused on electrically powered prostheses controlled by the residual limb’s muscle signals, termed myoelectric control [2]. These myoelectric devices utilize the existing neural pathways responsible for natural movement [3]. Although research in the area has focused on improving myoelectric control [4], studies continue to report rejection rates for electrically powered prosthetics as high as 35% in children and 23% in adults [5]. Another study showed that two of the most common reasons for rejection were poor functionality (98% of respondents) and a lack of sensory feedback (85% of respondents) [6].

Implementing exteroceptive sensory feedback into prosthetic devices is hypothesized to improve function by providing information about the prosthesis state to the user. The prosthetic hand’s grip force is a highly desired sensory signal for prosthesis users [7]. The measured grip force can be transferred to a prosthesis user’s residual limb, closing the loop during grasp control. Many different surface-mounted methods to translate the measured grip force back to the user have been investigated, including mechanotactile, vibrotactile, and electrotactile [8,9,10,11]. Researchers have observed promising results indicating improved prosthesis performance and embodiment when using these non-invasive feedback methods [12].

Recent advancements have allowed for somatotopically-matched grip force feedback by eliciting sensations in the missing fingers [9,13,14]. Persons with an amputation can experience non-painful sensations on the missing fingers when specific skin areas on the residual limb are stimulated, which constitute the so-called phantom hand map [15]. Targeted muscle reinnervation (TMR) is a well-established surgical procedure for improving prosthetic control by re-routing efferent nerves from the lost limb to nearby intact muscle groups [16]. This surgery also re-routes sensory nerve fibers that can reinnervate the surrounding skin, resulting in a restored hand map at the TMR location. Targeted sensory reinnervated (TSR) regions can be located away from reinnervated motor sites, making it possible to have both motor control and sensory feedback devices operating simultaneously [17]. Utilizing this technique, researchers were able to show more human-like prosthesis function when leveraging multiple modalities of feedback [18]. Recent advances in peripheral nerve interfaces allow electrodes to be surgically implanted directly into the peripheral nervous system for long term use [14,19,20]. Stimulation typically includes using either current modulated or voltage-modulated square wave pulse trains. The amplitude and frequency of these square waves can be adjusted to elicit various intensity and spatial selectivity [19,21]. This technique can produce sensations of touch, joint movement, and position [22]. However, these sensations’ level of naturalness has been subjectively stated as limited [23], although recent work has made substantial advances towards invoking a variety of distinct sensory percepts [24]. Current systems use multiple electrodes in a miniature array to increase stimulation channels [25,26,27]. Implanted peripheral nerve interfaces have been used to discriminate object size and stiffness [28]. They have also demonstrated functional performance increases in longitudinal studies [29,30,31,32] and stability in sensory percepts over long periods [21]. A recent study utilized implanted stimulation to deliver tactile and substituted proprioceptive feedback with high-performance results from transradial persons with amputations [25]. Despite recent studies trending towards utilizing somatotopically-matched feedback systems, the functional benefits of utilizing a feedback signal that is somatotopically-matched have not been compared to those of a non-somatotopically-matched feedback site.

The available techniques for somatotopically-matched feedback for persons with amputation result in spatial regions and sensations that vary in strength and modality uniquely for each individual. This individuality makes consistency between participants difficult in an experimental study. Additionally, not all persons with amputation have access to somatotopically-matched feedback sites, decreasing the number of available participants. A simulated prosthesis is a device that allows a non-disabled individual to emulate myoelectric control without a limb-amputation. Simulated prostheses are used to study myoelectric control strategies [33,34,35,36,37,38,39,40], myoelectric training techniques [41], and sensory feedback techniques [42,43]. Some of these devices have been shown to effectively reproduce myoelectric users’ performance metrics and motion kinematics [44]. With a simulated prosthesis, the participant pool includes non-disabled individuals, and the participant’s fingertip can be used as a site for somatotopically accurate feedback. All non-disabled individuals can use the same simulated prostheses, ensuring consistency between participants.

We developed an experimental protocol and suite of outcome measures for sensory-feedback-related myoelectric prosthesis research. This protocol consists of a standardized grasp and lift task with a modular apparatus suitable for adjusting various myoelectric prosthesis parameters. Many outcome measures related to myoelectric performance are outlined relating to task success, task execution time, and myoelectric strategy. We used this protocol to conduct a small pilot study investigating the functional myoelectric performance difference when using a somatotopically accurate feedback site over a non-somatotopically accurate feedback site. These preliminary findings revealed large between-participant effect sizes, showing that chosen outcome measures are sensitive to myoelectric performance. These results indicate that changing stimulation location of grip force feedback likely impacts myoelectric performance. The between-participant effect sizes were used to conduct a power analysis to inform a future larger study.

## 2. Materials and Methods

### 2.1. Modular Simulated Prosthesis

**Figure 1:**
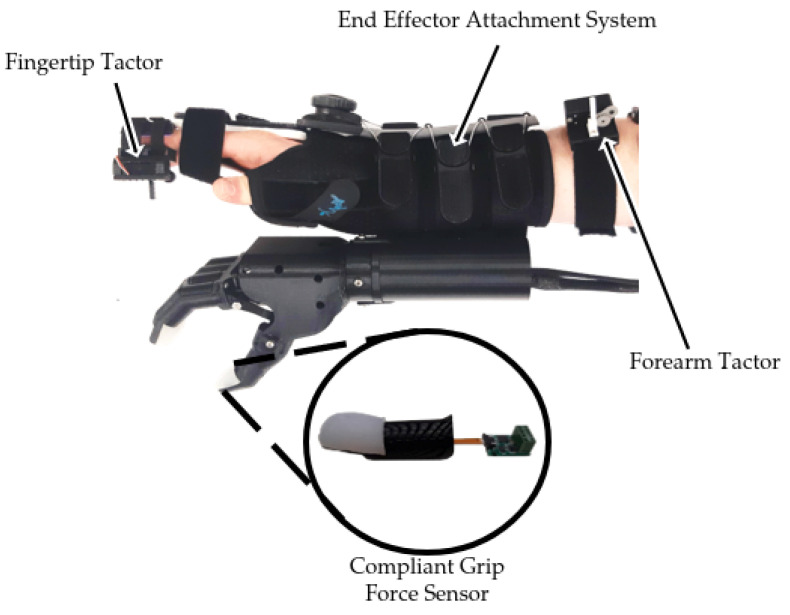
Modular simulated prosthesis adapted from [45].

**Figure 2:**
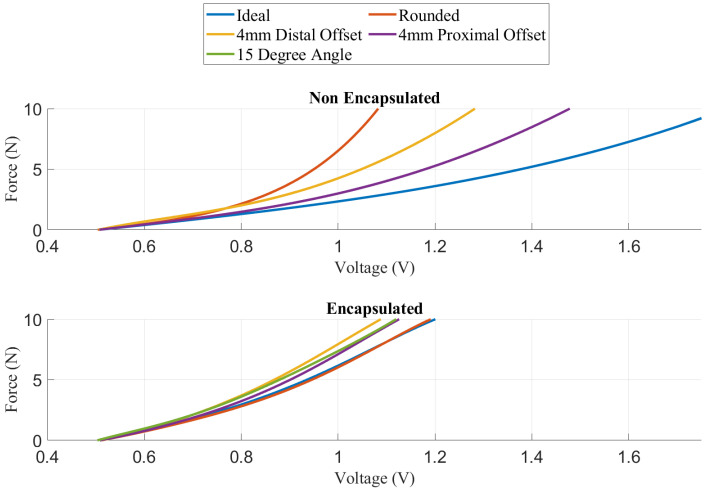
Summary of MSP grip force sensor accuracy under various loading conditions adapted from [45].

A modular simulated prosthesis (MSP) was previously developed by the authors with a focus on modularity, weight reduction, and comfort while allowing for mechanotactile sensory feedback to be applied to both the forearms and the fingertips [45]. An overview of the device is shown in Figure 1. The MSP has inexpensive capacitive force sensors (SingleTact, PPS UK Limited, Glasgow, United Kingdom) located on the index, middle, and thumb digits. These sensors are encased in a compliant silicon-based material, which allowed them to have up to 15 times improved accuracy in non-ideal loading conditions typical for prosthesis use [45]. An overview showing the loading response of the encapsulated fingertips versus the baseline non-encapsulated version is shown in Figure 2. Further details on the mechanical design of the feedback system and myoelectric hand are outlined in the following sections.

#### Mechanotactile Tactor Design

**Figure 3:**
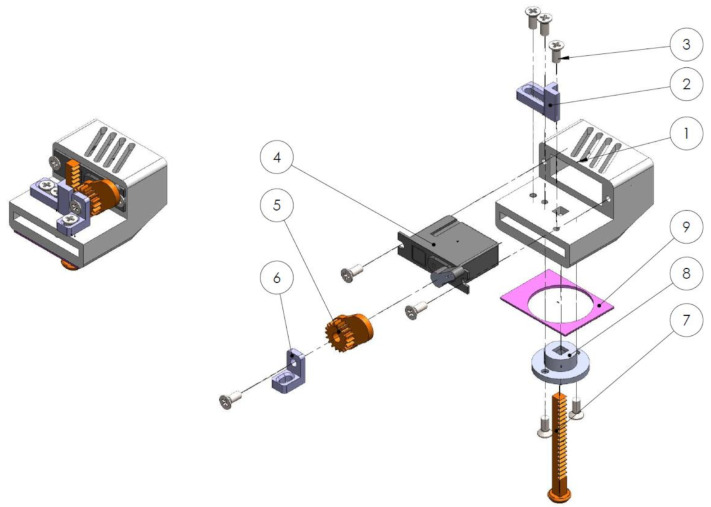
Mechanotactile tactor components (1) servo casing, (2) rack gear mount, (3) M2.5 × 5 mm screws, (4) D47 servo motor, (5) pinion gear, (6) pinion gear mount, (7) rack gear, (8) rack gear plug, (9) washable foam.

**Figure 4:**
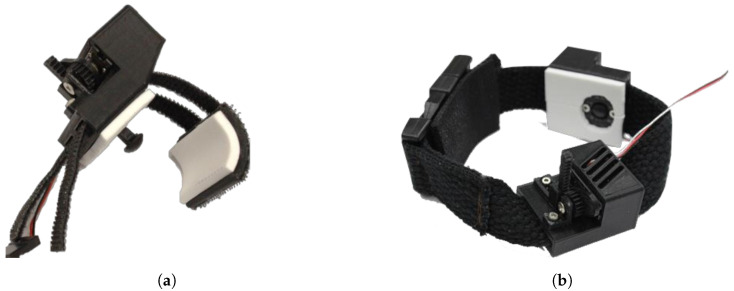
(**a**) Mechanotactile tactors on forearm mounting system. (**b**) Mechanotactile tactors on fingertip mounting system.

The mechanotactile tactors on the MSP utilize a lightweight Dymond D47 servo motor (Monroe, NC, USA) with a 3D-printed module M0.5 rack and pinion system. An 8 mm diameter rounded plunger head located on the lower end of the rack applies a linear force to the user. Slots were added on the rack gear mount to allow for adjustability of the center distance between the rack and pinion gear, ensuring a proper fit regardless of tolerance errors. A pinion mount was added to support the pinion gear along the rotation axis, reducing transverse forces on the servo motor shaft. A rack gear plug was placed to ensure no debris or fabric from mounting systems contacts the rack gear. The closed-cell foam was placed on areas where the material would contact the skin to allow for sanitization between participants. An overview of the device components is shown in Figure 3. Two mounting systems were developed to apply feedback to the fingertips, representing somatotopically accurate feedback, or the forearm, representing modality matched feedback. The tactor slides onto a non-stretchable band wrapped around the user’s forearm and is clipped in place for the forearm feedback. For the fingertip feedback, two hook Velcro straps wrap around the finger and adhere to a loop Velcro base located on the back of the fingertip. Both devices are shown in Figure 4. These tactors provide up to 12 N of force with a maximum amplitude of 14 mm.

### 2.2. End-Effector Design

**Figure 5:**
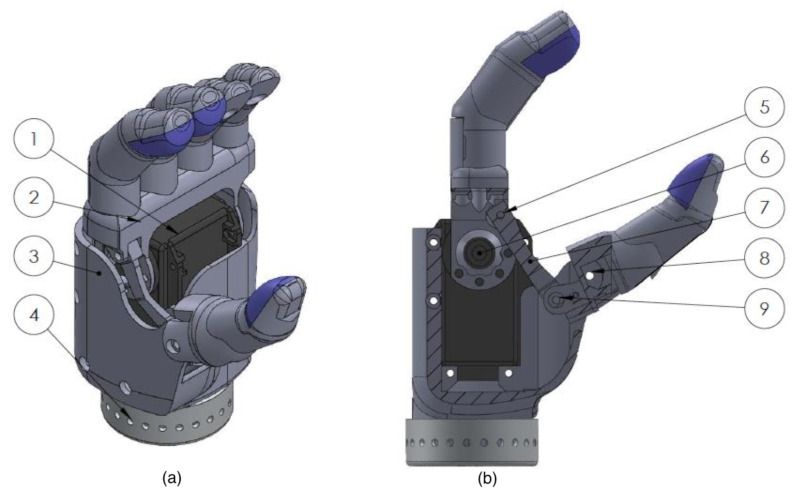
(**a**) Isometric view of the 3D-printed sensorized prosthetic hand. (**b**) Cutaway view of linked bar mechanism. (1) Dynamixel MX-64AT, (2) rigid finger brace, (3) hand casing, (4) wrist adapter, (5) linked bar finger rotation point (6) dynamixel rotation point, (7) linked bar mechanism (8) fixed thumb rotation point (9) linked bar thumb rotation point.

A 3D-printed PLA, anthropometric, single-degree-of-freedom (hand open, hand close) end effector was designed (Solidworks, 2018). This sensorized prosthetic hand is driven by a Dynamixel MX-64AT servo motor (Robotis, Inc., Seoul, South Korea) with the specifications listed in Table 1. The four fingers are rigidly connected and attach directly to the Dynamixel rotation point. The fingers and thumb are actuated simultaneously through a linked bar mechanism, as illustrated in Figure 5.

**Figure 6:**
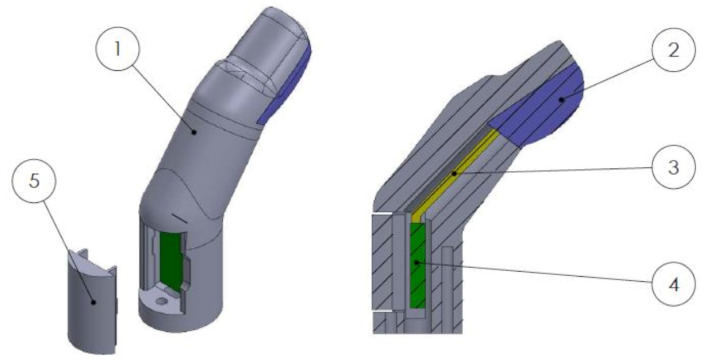
Exploded and section views of a finger with a compliant sensor. (1) PLA finger, (2) compliant sensor, (3) SingleTact wire, (4) ADC board, (5) snap-fit lid.

The previously presented compliant force sensors were added to the index finger, middle finger, and thumb of the end effector to measure grip force. The capacitive sensors require an attached analog-to-digital converter (ADC) located approximately 5 cm from the sensor pad. This ADC board was placed within the finger of the prosthetic hand. The fingers were hollowed out to create a cavity for the electronic circuits and connection wires. A snap-fit lid allows for installment and access to the embedded ADC box. The details of a compliant sensor within a prosthetic hand finger are shown in Figure 6. The compliant fingertip sensors created a more stable grasp by allowing three contact points, increasing contact surface area, and causing higher friction between the fingertips and the object.

### 2.3. Hand Restraint Mechanism

A wrist and thumb support brace (MedSpec, Orange, CA, USA) restrains the user’s hand to ensure isometric contraction during electromyography (EMG) control. This commercially available product was designed to be comfortable, lightweight, and adjustable; and leaves adequate space on the proximal forearm for EMG sensors and forearm mechanotactile tactors.

### 2.4. Fragile Object Simulator Device

**Figure 7:**
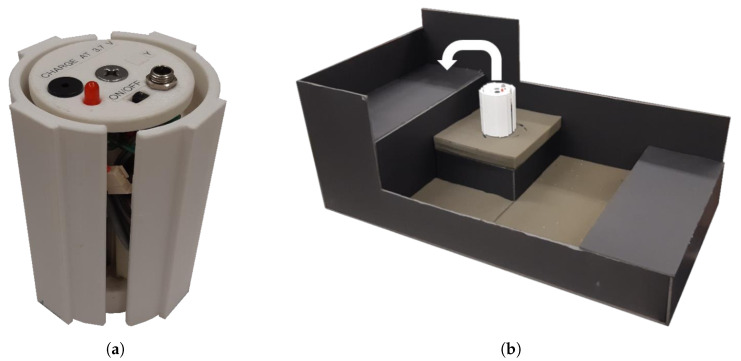
(**a**) Fragile object. (**b**) Experimental setup.

The fragile object was a 3D-printed (PLA) cylinder previously designed and presented in [46]. This device was instrumented with a 9-degree-of-freedom (DOF) inertial measurement unit (IMU) for orientation and acceleration measurement Figure 7a. Flat edges were designed along the cup’s edges to ensure consistent and repeatable contact of the MSP fingertips. The cup weighed 272 g, and with the compliant fingertips of the MSP took approximately 2 N of grip force to create enough friction to lift the fragile object without slipping. Note that the MSP’s maximum grip force value is 11 N. Preliminary testing indicated that a break force threshold value between 6.5 and 7.5 N would ensure that the average participant could complete the task with a success rate between 60 and 80%.

### 2.5. Study Participants

Three non-disabled individuals were recruited to participate in this study (2 females; age: 34 ± 9 (mean ± SD); 1 ambidextrous). Participants will be referred to as PID1, PID2, and PID3. Participants had no upper limb dysfunction, i.e., no muscular or neurological dysfunction. PID1 had previous experience operating a simulated prosthesis. The experiment was conducted in a single 3 h long session. Written informed consent according to the University of Alberta Health Research Ethics Board (Pro0007893) was obtained from all participants before conducting the experiment.

### 2.6. Experimental Setup

Participants were fit with the MSP, with the grip force fingertip measurement and mechanotactile tactors described previously. Participants used noise-canceling headphones to ensure no sound from the MSP or tactor motors could be used as incidental feedback. There were two feedback location conditions: finger and forearm. For the finger feedback condition, tactors were placed on the thumb and index fingers of the participant. The thumb tactor was mapped to the grip force sensor in the thumb of the MSP. The index tactor was mapped to the sum of the grip force sensor in the MSP index and middle finger. This mapping allowed for the utilization of three contact points of compliant digits when grasping the fragile object while maintaining sensor accuracy. For the forearm feedback condition, two tactors were placed approximately 10 cm apart on the forearm’s volar surface. This positioning is similar to a study conducted previously on mechanotactile discrimination [47] and ensures that the tactors are further apart than the noted two-point discrimination distance on the forearm of 38 mm [48,49]. A protective sensation test was conducted with a 10 g monofilament to ensure that each participant had a normal sensation in their forearm before beginning the experiment. Two-point discrimination tests were conducted to ensure the participant could discriminate between the two tactor positions.

The MSP’s control was calibrated by monitoring eight EMG signals from a Myo Armband (Thalmic Labs, Inc., Kitchener, ON, Canada) placed on the participant’s upper forearm (proximal to the forearm tactors) while the participant underwent a series of wrist flexion and extension isometric muscle contractions. The two channels with the highest activation were mapped to open and close the hand. This type of control is also known as conventional myoelectric control. Special care was taken to ensure that additional weight from lifting an object did not result in EMG activation of the two chosen electrodes. This lift activation check was done by monitoring the signals while placing a vertical load on the end-effector and adjusting the minimum and maximum thresholds and gains accordingly.

### 2.7. Experimental Protocol

The experimental protocol consisted of 4 blocks corresponding to the four feedback conditions. The first and third block for all participants was a no-feedback condition. The second and fourth block contained either the arm or finger feedback condition, depending on which presentation order the participant underwent. Each block consisted of 4 phases: general object manipulation training, fragile object manipulation training, task familiarization phase, and task testing phase.

During the general object manipulation training phase, participants were allowed a 3 min training segment to practice using the MSP system. This phase consisted of grasping and lifting various objects such as a foam ball and a stackable cup. The following fragile object manipulation training phase lasted for 2 min, which was dedicated to manipulating the fragile object. The fragile object was set to administer the break sound feedback each time the break threshold was exceeded, allowing the participant to gain familiarity with the system. Verbal instructions were given to ensure the participants were performing similar motions during the training segment.

**Figure 8:**
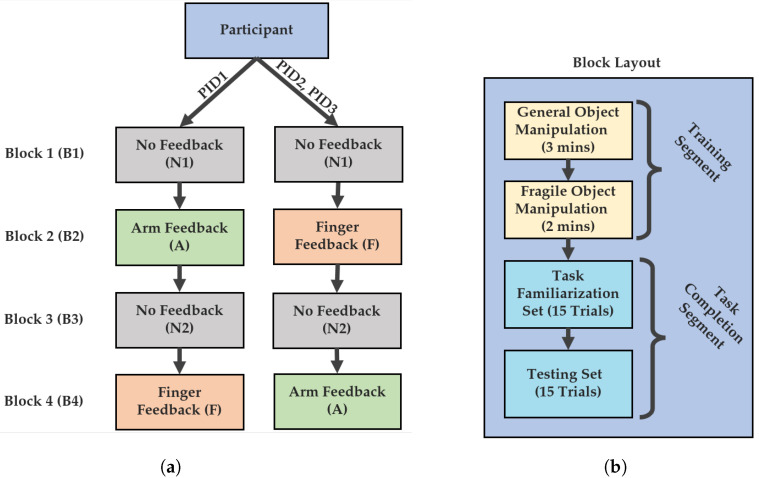
Experimental protocol. (**a**) Block presentation order summary. (**b**) Block layout summary.

After the training phases, the task familiarization phase commenced. The familiarization phase consisted of a set of 15 trials. During that phase, participants were instructed to move the fragile object from the starting position to the 10 cm high shelf position with the lightest grasp force possible in less than 20 s. Participants were instructed to focus on grasping lightly rather than task speed, as 20 s was more than enough time to complete the task (only one trial of all participant trials went overtime). The task setup can be seen in Figure 7b. The last phase, task testing phase, was identical to the task familiarization phase. The outcome measures were calculated using only the task testing phase trials, similarly to previous work [50]. Using only the testing set for calculation was done to reduce any confounding learning effects throughout the task completion segment. Participants were given mandatory 1 minute rest periods after the training phases and after each of the testing phases. Additional breaks were provided whenever requested by the participants. An overview of the protocol and the order of feedback presentation is shown in Figure 8.

The fragile-object-breaking threshold for the first participant was set to 6.5 N rather than 7.5 N. Although the first participant still had a success rate in the desired range, he was an experienced myoelectric user. The following two participants were not experienced myoelectric users, and so the threshold was raised to 7.5 N to ensure enough successful trials occurred. Additionally, the first participant completed fewer trials (20 for each feedback condition) than the other participants. The number of trials per block was raised from 20 to 30 to increase the number of trials available for data analysis. For the first participant, the task completion segment was split into 10 trial sets rather than 15 trial sets. However, all metrics used were calculated within-participant, so this participant’s data were still included for this pilot study.

Data were collected at 50 Hz from the tactor system, fragile object, and the MSP through three independent GUIs (C#, Visual Studio Express 2015 (Microsoft, Redmond, WA, USA)). The tactor system automatically logged grasp force from each digit, tactor positions, and timestamps. The fragile object automatically logged quaternion information representing orientation; raw accelerometer values in the x, y, and z directions; and timestamps. The brachI/Oplexus software (BLINC lab, Edmonton, AB, Canada) [51] automatically logged all 8 EMG signals; and the MSP hand position, velocity, torque, temperature, current draw, and timestamps.

#### 2.7.1. Outcome Measures

Selected outcome measures included success rate, maximum grasp force, completion time, grasp time, and hand aperture adjustments.

The repeated-measures experimental design allowed each participant to act as their own baseline or control, reducing the impact of inherent variation between-participants by allowing for relative improvements to be analyzed.

##### Success Rate

A trial was deemed successful if the participant transferred the fragile object to the shelf without breaking or dropping it within the 20 s time limit. The ratio of successful trials to total trials in a testing set is the block’s success rate. This metric indicates task performance consistency. The added information provided by the feedback was hypothesized to result in an improved success rate for both feedback conditions.

##### Maximum Grasp

The largest value measured by the fingertip force sensors on each trial was recorded as the maximum grasp force, similarly to previous work [46,50]. The average maximum grasp for each block was calculated from all successful trials in the testing set to provide a single value for each block. A lower value indicates better grasp force control, as the participants were explicitly told to grip the cup as lightly as possible. The added information provided by the feedback was hypothesized to reduce the maximum grasp force for both feedback conditions.

##### Completion Time and Grasp Time

The time taken from task start to object release was defined as the completion time. The average task completion time for each block was calculated from all successful trials in the testing set to provide a single value for each block. The time taken from first contacting the object to first lifting the object was defined as grasp time. The average grasp time was calculated from all successful trials in each set to provide a single value for each feedback condition. The added information provided by the feedback was hypothesized to increase the completion and grasp time for both feedback conditions, as the participants would be more focused on the task.

**Figure 9:**
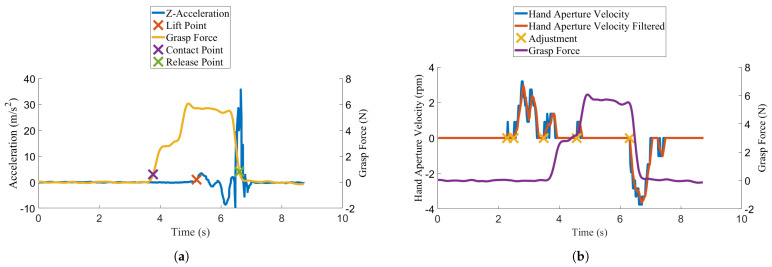
Outcome measure extraction (**a**) contact point, lift point, and release point (**b**) adjustments.

The contact point was calculated as the first instance where the grasp force exceeded 0.3 N. The lift point was defined when the z-acceleration of the fragile object first exceeded 1 m/s. The release point was calculated as the first point where the grip force fell below 0.3 N after the lift point. An example of these points extracted from a successful trial is shown in Figure 9a. The Xs indicate the contact point, lift point, and release point in a typical successful trial. Grasp force increases just before the defined contact point at around 3.8 s. After the grasp force increases to a stable level just after 5 s, the lift point is identified by an initial increase in z-acceleration. As the object is released, the grasp force decreases, marking the release point.

##### Hand Aperture Adjustments

A hand aperture adjustment was defined as a direction change in the prosthesis hand open/close velocity. The calculation of this value becomes problematic when the participant’s EMG signal is very close to the minimum motion threshold. The noise in the EMG signal results in the hand aperture velocity signal oscillating over the minimum threshold, creating artificial zero crossings in the hand aperture velocity at a frequency much faster than is capable by a human. The hand velocity signal was low-pass filtered using a moving average filter designed to have −3 dB of attenuation at 7 Hz to mitigate these motion artifacts. Seven Hertz was chosen, as it represents the average frequency exerted by humans during a fast tapping motion [52]. Seven Hertz is a conservative estimate of what gesture frequency a human could generate, since participants in the current study were activating wrist flexion and extension rather than fast finger motion. The moving average filter was chosen over other filtering techniques, since it does not cause signal overshoot, which could manifest as artificial motion changes upon the signal returning to zero. An example of the adjustments extracted from a successful trial is shown in Figure 9b. A positive hand velocity corresponds to the hand-close direction. The Xs indicate that an adjustment was made (i.e., a direction change of the prosthesis hand aperture velocity). Around the 3.5 s mark, the unfiltered hand aperture velocity can be seen to oscillate rapidly. However, this oscillation only results in one adjustment after the signal is filtered, as described. The hand adjustment metric was chosen to provide insight into the impact of feedback condition on grasp modulation during the fragile object task. The total number of adjustments from task start to object release was calculated from all successful trials in each set to provide a single metric for each feedback condition. The number of adjustments was hypothesized to increase for both feedback conditions due to an increased focus on the task.

#### 2.7.2. Statistical Analysis

In this paper, the following labeling convention was used. The "A" label represents the arm feedback, "F" represents the finger feedback, and Navg represents the average between the two no-feedback conditions. The A-N label is used to indicate the difference between the arm feedback condition and Navg. The F-N label is used to indicate the difference between the finger feedback condition and Navg. Additionally, the F-A label represents the difference between the finger feedback condition and the arm feedback condition. To clarify, the second abbreviation is subtracted from the first abbreviation; for example, a positive A-N value indicates that the arm condition had a higher value than the Navg condition.

In this study, individual effect sizes were computed on a per-participant basis to analyze each participant separately for the A-N, F-N, and F-A comparisons. These individual effect sizes were calculated using the mean difference and pooled standard deviation between both conditions being compared for each participant separately. Effect sizes were calculated using Cohen’s D, which compares two means [53].

Between-participant effect sizes were computed using the group mean difference and standard deviation of difference scores for the A-N, F-N, and F-A comparisons. Using the between participant effect sizes, a power analysis was conducted to determine the required number of participants that would be required for a future study. G*Power [54] statistical analysis software was used to compute the power analysis with a two-tailed one-sample *t*-test, an α-level of 0.05, and a β-level of 0.8.

## 3. Results

### 3.1. Within-Participant Results

**Figure 10:**
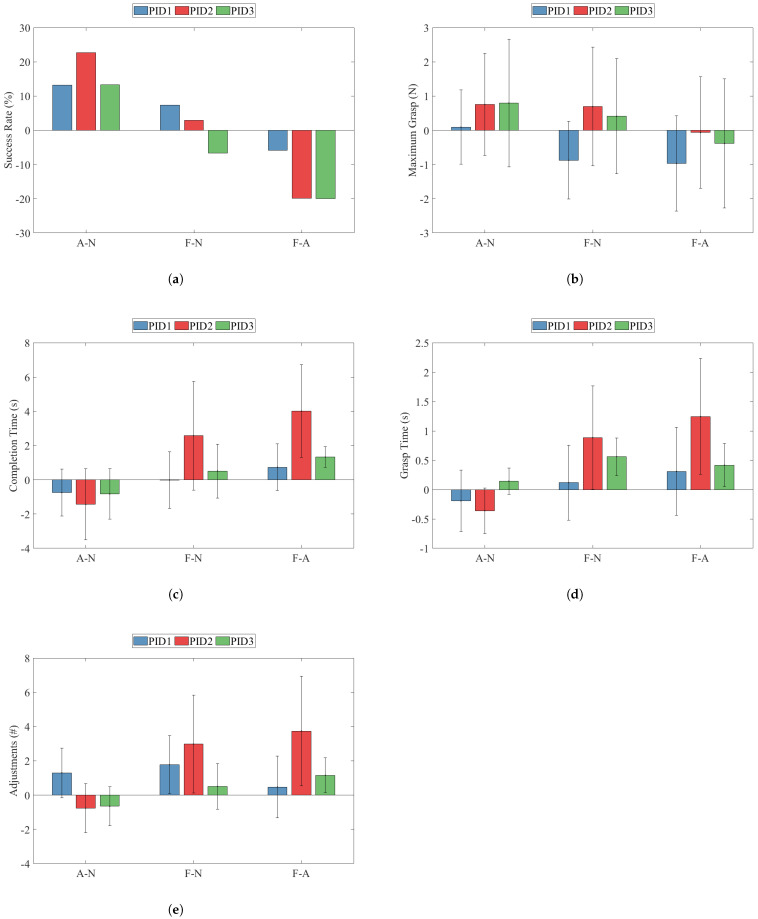
Within-participant results for (**a**) success rate, (**b**) maximum grasp, (**c**) completion time, (**d**) grasp time, (**e**) adjustments.

All the within-participant comparison results can be seen in Figure 10. Each error bar represents the pooled standard deviation of a metric. Note that the success rate metric was calculated using all trials from each set; therefore, error bars are not present for it. For all metrics excluding success rate, effect sizes were computed per participant using their individual mean and standard deviation. The effect sizes are summarized in Table 2.

#### 3.1.1. Success Rate

The A-N comparison’s success rate between all participants had a minimum of 13.2% (PID1) and a maximum of 22.7% (PID2). All participants showed an increase in the success rate upon receiving the arm feedback condition compared to the no-feedback condition. The F-N comparison’s success rate was much less consistent, having values ranging from −6.7% (PID3) to 7.3% (PID1). Interestingly, PID3 decreased in performance upon receiving the finger feedback. The F-A values show that the finger feedback condition resulted in lower success rates than the arm feedback condition for all participants.

#### 3.1.2. Maximum Grasp

All participants showed a higher average maximum grasp upon receiving the arm feedback condition than the no-feedback condition. PID2 and PID3 showed moderate increases of 0.76 N (d = 0.51) and 0.80 N (d = 0.42), whereas PID 1 had a very small increase iof about 0.10 N (d = 0.08). The F-N values for maximum grasp were much less consistent between participants. Again, PID2 and PID3 showed increases in maximum grasp of 0.69 N (d = 0.39) and 0.41 N (d = 0.25). PID1 was the only participant to decrease maximum grasp, having a value of −0.87 N and a relatively large effect size of −0.70. The F-A values revealed that all participants displayed less maximum grasp force when interfacing with the finger feedback instead of the arm feedback. PID2 and PID3 had very small differences of −0.06 N (d = −0.04) and −0.39 N (d = −0.2), and PID1 had a higher difference of −0.97 N and a relatively large effect size of −0.69. PID1’s high myoelectric experience could have resulted in leveraging the finger feedback without losing control of the grasp force.

#### 3.1.3. Completion Time

All participants had a shorter completion time when receiving the arm feedback condition than the no-feedback condition: A-N values were −0.7 s, −1.4 s, and −0.82 s for PID1, PID2, and PID3. All participants had similar medium-to-large negative effect sizes of −0.57, −0.7, and −0.64 for PID1, PID2, and PID3. The finger feedback to no feedback comparison generally revealed opposite results. PID2 showed a large increase in the completion time of 2.6 s with a large effect size of 0.77. PID3 showed a smaller increase in 0.5 s with a moderate effect size of 0.38. PID1, however, showed no change with an effect size of approximately zero. The opposite effects of the arm and finger feedback conditions were highlighted by the F-A metric: all participants showed higher completion time values for the finger feedback condition. PID1 was the least affected by feedback location, as shown by a F-A value of 0.7 s and a moderate effect size of 0.54. PID2 had a much larger change in the completion time of 4.0 s, with a very large effect size of 1.49. PID3 had a smaller change in the completion time of 1.33 s; however, the small standard deviation led to an extremely large effect size of 2.18. This value was the largest within-participant effect size for any metric.

#### 3.1.4. Grasp Time

PID1 spent slightly less time in the grasp phase during the arm feedback condition than the no-feedback condition, by −0.19 s, with a moderate effect size of −0.36. PID2 also spent slightly less time in the grasp phase during the arm feedback condition than the average no-feedback condition, by −0.36 s; however, the effect size was quite large at −0.94. PID3 showed the opposite trend for the A-N metric by a small increase in grasp time of 0.14 s with a moderate effect size of 0.51. All participants showed an increase in grasp time during the finger feedback condition compared to the no-feedback condition. PID2 showed the largest increase of 0.89 s, with a large effect size of 0.88. PID3 showed an increase in grasp time of 0.56 s and a very large effect size of 1.51. PID1 showed the lowest increase in grasp time of 0.12 s and a small effect size of 0.16. Again, the difference between the finger feedback and arm feedback condition for grasp time was highlighted by the F-A metric; all participants showed a positive value. PID2’s grasp time was 1.00 s higher for the finger feedback condition, with a very large effect size of 1.27. PID3’s grasp time was 0.56 s higher for the finger feedback condition, with a large effect size of 1.13. PID1 showed a smaller change of only 0.12 s for the finger feedback with a small effect size of 0.41.

#### 3.1.5. Adjustments

The A-N comparison for adjustments showed that PID1 increased the number of adjustments by 1.3 during the arm feedback condition compared to the average no-feedback condition with a large effect size of 0.89. PID2 and PID3 showed small decreases in adjustments of −0.8 and −0.7, with corresponding moderate effect sizes of −0.53 and −0.63. All participants agreed for the F-N metric, there being more adjustments for the finger feedback condition than the average no-feedback condition. PID1 and PID2 showed increases of 1.8 and 3.0, with large effect sizes of 0.97 and 0.92, respectively. PID3 had a much smaller increase in adjustments of 0.5, with a small-to-moderate effect size of 0.39. The F-A values for adjustments were positive for all participants, showing more adjustments during the finger feedback condition than the arm feedback condition. PID1 showed a very small difference of 0.5 with a small effect size of 0.26. PID2 showed a much larger difference of 3.7 with a large effect size of 1.17. PID3 showed a difference of 1.13, also with a large effect size of 1.14.

### 3.2. Between-Participant Power Analysis

Between-participant effect sizes and the corresponding estimated numbers of required participants computed using the described power analysis are shown in Table 3.

The largest between-participant effect size seen was for the A-N comparison’s success rate, requiring an estimated three participants to achieve statistical significance. Since this study had three participants, this result was statistically significant (t = 6.35, *p* = 0.024). The between-participant effect size for the F-N comparison of success rate was very small: the estimated requirement was over 100 participants to achieve statistical significance. The F-A comparison for success rate resulted in a large negative between-participant effect size, requiring an estimated four participants to achieve statistical significance. Results were similar for the maximum grasp, requiring an estimated 5 participants to obtain significance for the A-N comparison, 8 participants for the F-A comparison, and over 100 participants for the F-N comparison. Completion time also yielded high between-participant effect sizes, requiring an estimated 4 participants to achieve significance for the A-N comparison, 7 participants for the F-A comparison, and 12 participants for the F-N comparison. Grasp time showed a large estimated sample of 21 participants required for the A-N comparison and only 6 participants for the F-N and F-A comparison. Finally, the A-N comparison adjustments had an effect size of almost zero, and an estimated requirement of much more than 100 participants to achieve significance. However, the F-N and F-A comparisons had high between-participant effect sizes, resulting in estimated 5 and 8 participants needed to reach significance.

## 4. Discussion

In this work, we developed an experimental protocol and compiled a suite of defined outcome measures to standardize testing between studies that aim to utilize sensory feedback for upper-limb prosthesis users. We utilized this protocol to investigate the effect of mechanotactile feedback stimulation location on myoelectric prosthesis performance. Our results from the pilot study presented in this work may be used to inform future studies on the role of feedback stimulation location on prosthesis use.

### 4.1. Success Rate

The within-participant success rate values showed the efficacy of the arm feedback condition over no feedback. Even in this small pilot study, statistical significance was obtained for this metric. The range of success rate increase values for the arm feedback condition over the no-feedback condition (13.2% to 22.7%) was consistent with the literature. Meek et al. conducted a similar myoelectric fragile object transfer task with mechanotactile feedback delivered to the forearm, which resulted in a success rate increase in 5–20% [55]. Although exact values were not reported, Clemente et al. found success rate increases of up to 6% using discrete vibrotactile feedback delivered to the upper arm on a similar task [56]. The consistency of success rate with the literature demonstrates the MSP’s efficacy as an experimental platform. The finger feedback did not appear to benefit the success rate over the no-feedback condition. Consequently, when compared directly with the arm feedback condition, the finger feedback condition had a much lower success rate with a very large effect size of −2.29. From this pilot study, the success rate appears to be affected by the sensory feedback location.

### 4.2. Maximum Grasp Force

Within-participant effect sizes for average maximum grasp force were small due to the large variances in each testing set. However, the average maximum grasp difference values between conditions were consistent for all participants. The consistency in difference values produced large between-participant effect sizes, indicating that the arm feedback condition resulted in higher grasp forces than the no-feedback and finger feedback conditions. However, all within-participant difference values were less than 1 N in magnitude, representing about 10% of the MSP’s maximum grasp force. Although the MSP’s compliant fingertip sensor had a resolution of 0.03 N, the system’s noise was 0.3 N due to the long cables required for mobility. The small within-participant values were of a similar magnitude to the sensor noise and small compared with the variance for any participant on any individual block. This result was reflected in a previous study, where the maximum value of generated forces for a myoelectric target acquisition task did not change significantly with added feedback, although this study was done with discrete vibrotactile feedback [50]. Other studies have reported a much larger decrease in grasp force magnitude when presented with sensory feedback during fragile object transfer tasks for proportional mechanotactile and proportional vibrotactile feedback. Kim et al. found that maximum grasp decreased between 28% and 43% when mechanotactile feedback was administered to TMR sites [57]. Pylatiuk et al. found that the grasp force decreased by an average of 54% when mechanotactile feedback was provided to the upper arm of five persons with amputation [58]. Schoepp et al. similarly found a maximum grasp force decrease in 21% when mechanotactile feedback was provided to the upper arm of a person of amputation [46]. It is hypothesized that the lack of fine control in this pilot study was due to the MSP grasp force changing too quickly for the participants to react. The delay from grasp force measurement to tactor actuation, in addition to human reaction time, was likely too long for participants to use the feedback for fine grasp control.

### 4.3. Completion Time and Grasp Time

General trends of decreased completion time and decreased grasp time were observed for the arm feedback condition over the no-feedback condition, while the opposite was observed for the finger feedback condition. A recent study found an increase of 13% in task completion time when implanted electrotactile feedback was presented [29]; however, while this feedback’s perceived location was in the missing limb, the perceived modality was not reported. In another study, proportional somatotopically-matched feedback, similar to the finger feedback case, also increased task completion time [57]. Another study with proportional modality matched feedback, similar to the arm feedback case, showed an increase in task completion time, contrary to this pilot study [46]. In contradiction, Meek et al. found a decrease in completion time for the same feedback strategy [55]. Clemente et al. found no change in task completion time using discrete vibrotactile feedback on a fragile object task [59]. Different values reported throughout the literature show the experimental setup’s impact on the task timing outcome measures. The comparisons made in this study between the arm feedback condition and the finger feedback condition are more informative about the effect of feedback location due to the data being recorded with the same apparatus, with the only altered variable being feedback location. The finger feedback condition resulted in higher completion time and grasp time for all participants than the no-feedback condition and the arm feedback condition. The large within-participant effect sizes were from 0.54 to 2.18 for completion time and 0.41 to 1.27 for grasp time. This consistently slower movement was likely caused b increased focus on the task with the more sensitive finger feedback site. The between-participant effect sizes for the finger feedback to arm feedback comparison produced for both completion time and grasp time were large, requiring an estimated seven participants to obtain statistical significance. These results indicate that both completion time and grasp time are sensitive outcome measures to the somatotopic accuracy of feedback.

### 4.4. Adjustments

The hand aperture adjustments outcome measure was newly introduced in this study. It showed mixed results for the arm feedback condition compared to the no-feedback condition, with two participants decreasing the number of adjustments and one participant increasing it. The finger feedback condition had more adjustments than the no-feedback and arm feedback conditions for all participants. A recent study showed that participants adjusted their control signals significantly more when given continuous audio feedback than when given discrete vibrotactile feedback delivered to the forearm [60]. Although the audio feedback used myoelectric control as an input, the information could be considered richer than that of the vibrotactile, which only output a short burst during discrete events. The rich audio feedback could be likened to the finger feedback in this study, conveying more information than the arm feedback condition due to the increased sensitivity at the fingertip. The proposed total adjustments outcome measure proved sensitive to the somatotopic accuracy of feedback. Large between-participant effect sizes were observed between finger feedback and both no feedback and arm feedback. The power analysis revealed that a participant pool of eight would be required to obtain statistical significance for this metric. While this metric does not directly relate to functionality, it provides insight into how the participant uses the prosthesis that may not be easily seen through other outcome measures.

## 5. Conclusions

Despite many advancements related to myoelectric prostheses, rejection rates remain high [6]. A lack of sensory feedback is commonly cited as a reason for rejection [5]. Recent research focused on achieving more natural sensory perceptions felt in the missing digits, allowing for somatotopically-matched feedback systems [14]. The functional impact of utilizing a somatotopically-matched feedback site over a non-somatotopically-matched feedback site has not been previously investigated.

A pilot study was conducted to compare modality matched mechanotactile grip force feedback in somatotopically-matched and non-matched conditions during a fragile object transfer task. A range of effect sizes for a variety of performance outcome measures were calculated. All participants showed similar difference values in the comparison between the finger feedback condition and the arm feedback condition, creating large between-participant effect sizes for all outcome measures. This within-participant consistency highlights the importance of the repeated measures design. The arm feedback’s success rate was higher than that of the finger feedback. This decreased success rate of the finger feedback condition could be potentially attributed to the finger’s higher sensitivity resulting in an increased focus on the feedback rather than control of the prosthesis. The completion times, grasp times, and adjustments by all participants during the finger feedback test were substantially higher than in the arm feedback test, indicating that more attention was on the task. A lack of fine control over the maximum grasp force was exhibited by all participants, characterized by the considerable variation during all feedback conditions. This variation was hypothesized to be caused by the MSP’s grasp force increasing quicker than the participants’ reaction time. For future studies, the prosthesis control method could be modified to decrease the speedat which the MSP applies grip force, although this may not represent real-world prosthetic hand speeds.

These preliminary results indicate that the feedback location could play a role in prostheses control. The power analysis revealed that an estimated participant pool of *n* = 8 would be enough to achieve significance for comparing the arm and finger feedback conditions for all proposed metrics. However, due to this pilot study’s low recruitment, the 95% confidence intervals of the between-participant effect sizes are considerable.

The difference between the arm feedback’s success rate and the no-feedback’s success rate was comparable to the values reported in the literature, lending credibility to the MSP as a standardized experimental apparatus. This pilot study showed the feasibility of the proposed experimental task and confirmed sensitive outcome measures. Data analysis techniques were also established that would scale to a more extensive study. Quantifying the performance effects of utilizing a somatotopically-matched feedback site will be critical in future evaluations of myoelectric prosthesis feedback systems.

## Figures and Tables

**Table 1:** 3D-printed prosthetic hand specification summary.

Mass (g)	298
Maximum Grip Aperture (mm)	125
Maximum Grip Strength (N)	11
Degrees of Freedom	1
Maximum Grip Speed (deg/s)	180
Maximum Current Draw (A)	1.0 (@40% power)
Operating Voltage (V)	12
Cost ($ CAD)	550

**Table 2:** Effect sizes for relative within-participant results (green shading represents a positive value, red shading represents a negative value, strength of shading represents the magnitude of value.

	Maximum Grasp			Completion Time			Grasp Time			Adjustments		
	A-N	F-N	F-A	A-N	F-N	F-A	A-N	F-N	F-A	A-N	F-N	F-A
**PID1**	0.08	−0.7	−0.69	−0.57	−0.01	0.54	−0.36	0.16	0.41	0.89	0.97	0.26
**PID2**	0.51	0.39	−0.04	−0.7	0.77	1.49	−0.94	0.88	1.27	−0.53	0.92	1.17
**PID3**	0.42	0.25	−0.2	−0.64	0.38	2.18	0.64	1.51	1.13	−0.63	0.39	1.14

**Table 3:** Between-participant power analysis summary (green shading represents a positive value, red shading represents a negative value, strength of shading represents the magnitude of value.

	Effect Size			Required Participants for Significance		
	A-N	F-N	F-A	A-N	F-N	F-A
**Success Rate**	3.67	0.20	−2.29	3	>100	4
Maximum Grasp	1.70	0.11	−1.26	5	>100	8
Completion Time	−3.24	0.92	1.42	4	12	7
Grasp Time	−0.65	1.65	1.57	21	6	6
Adjustments	−0.04	1.74	1.27	>100	5	8

## Data Availability

The data presented in this study are available upon request to the corresponding author.
